# A reflection on invasive pneumococcal disease and pneumococcal conjugate vaccination coverage in children in Southern Europe (2009–2016)

**DOI:** 10.1080/21645515.2016.1263409

**Published:** 2016-12-20

**Authors:** Marta Moreira, Olga Castro, Melissa Palmieri, Sofia Efklidou, Stefano Castagna, Bernard Hoet

**Affiliations:** a GSK Vaccines, Global Medical Affairs, Wavre, Belgium; b GSK, Medical Affairs Vaccines, Algés, Portugal; c GSK, Medical Affairs Vaccines, Madrid, Spain; d GSK, Medical Affairs Vaccines, Athens, Greece; e GSK, Medical Affairs Vaccines, Verona, Italy

**Keywords:** invasive pneumococcal disease, national immunisation programmes, pneumococcal conjugate vaccine, Southern Europe, vaccination coverage

## Abstract

Higher-valent pneumococcal conjugate vaccines (PCVs) were licensed from 2009 in Europe; similar worldwide clinical effectiveness was observed for PCVs in routine use. Despite a proven medical need, PCV vaccination in Southern Europe remained suboptimal until 2015/16.

We searched PubMed for manuscripts published between 2009 and mid-2016. Included manuscripts had to contain data about invasive pneumococcal disease (IPD) incidence, or vaccination coverage with higher-valent PCVs. This review represents the first analysis of vaccination coverage and impact of higher-valent PCVs on overall IPD in Southern European countries (Portugal, Spain, Italy, Greece, Cyprus).

Vaccination coverage in the Portuguese private market peaked around 2008 at 75% (children ≤ 2 years) but declined to 63% in 2012. In Madrid, coverage was 95% (2007–2012) but dropped to 67% (2013/14; children ≤ 2 years) after funding termination in May 2012. PCVs were recently introduced in the national immunisation program (NIP) of Portugal (2015) and Spain (2015/16). In Italy, coverage for the complete PCV schedule (children ≤ 2 years) was 88% in 2013, although highly variable between regions (45–99%). In Greece, in 2013, 82.3% had received 3 PCV doses by 12 months, while 62.3% received the fourth dose by 24 months. Overall IPD (net benefit: effect on vaccine types, vaccine-related types, and non-vaccine types) has decreased; in Greece, pneumococcal meningitis incidence remained stable.

Continued IPD surveillance or national registers using ICD-10 codes of clinically suspected IPD are necessary, with timely publicly available reports and adequate national vaccination registers to assess trends in vaccination coverage, allowing evaluation of PCVs in NIPs.

## Introduction

The first pneumococcal conjugate vaccine (PCV7; Prevenar/Prevnar™, Pfizer, New York, NY, USA) was licensed in 2000, and contains capsular polysaccharide of serotypes 4, 6B, 9V, 14, 18C, 19F, and 23F. Two new higher-valent PCVs were licensed in the European Union in 2009 and 2010. The pneumococcal non-typeable *Haemophilus influenzae* (NTHi) protein D conjugate vaccine (PHiD-CV; Synflorix™, GSK, Belgium) contains capsular polysaccharide of serotypes 1, 4, 5, 6B, 7F, 9V, 14, and 23F conjugated to NTHi protein D, of serotype 18C conjugated to tetanus toxoid, and of serotype 19F conjugated to diphtheria toxoid. The other higher-valent vaccine, PCV13 (Pfizer; Prevenar 13/Prevnar 13™, New York, NY, USA) contains capsular polysaccharide of serotypes 1, 3, 4, 5, 6A, 6B, 7F, 9V, 14, 18C, 19F, 19A, and 23F, with each polysaccharide conjugated to the CRM_197_ diphtheria carrier protein.

Even though the 2 new PCVs do not contain the same pneumococcus capsular antigens in their composition, similar impact of both vaccines against overall IPD (net benefit i.e. effect on vaccine types [VT], vaccine-related types [VRT], and non-vaccine types [NVT])^1^ and vaccine-type IPD, has been observed in post-marketing studies.^2-4^ Despite a proven medical need and evidence of public health benefit, PCV vaccination in Southern Europe remains suboptimal. This suboptimal vaccination coverage may be due to various potential barriers, such as the cost of the vaccines and their pharmacoeconomic evaluation, potential logistic issues, low disease awareness due to a lack of investment in National Surveillance Centres to support public and reliable data, or insufficient political prioritization to introduce PCVs. Vaccine hesitancy or refusal by the parents cannot be ruled out as a factor, although this is not likely to be one of the largest contributors of suboptimal vaccination coverage in Southern European countries. Vaccine hesitancy was mentioned as a cause of decreasing coverage in 2012–2014 for several vaccines in Italy, but PCV coverage had decreased by only 0.4%.^5^ In Portugal, vaccine hesitancy is not reflected in vaccination coverage rates, and adherence of the population to the NIP was mentioned as one of the reasons for the high coverage rates.^6^

This review represents the first analysis of the vaccination coverage and impact of higher-valent PCVs, focusing on overall IPD (net benefit) in Southern European countries. The aim is to provide an overview of published PCV vaccination coverage in Southern European countries, as well as the incidence of overall IPD in children less than 5 y. We focused on Portugal, Spain, Italy, Greece, and Cyprus, because challenges due to the economic crisis in Europe since 2008 and the related austerity measures in these countries may have an impact on public health.^7^

## Results

**Figure 1.**
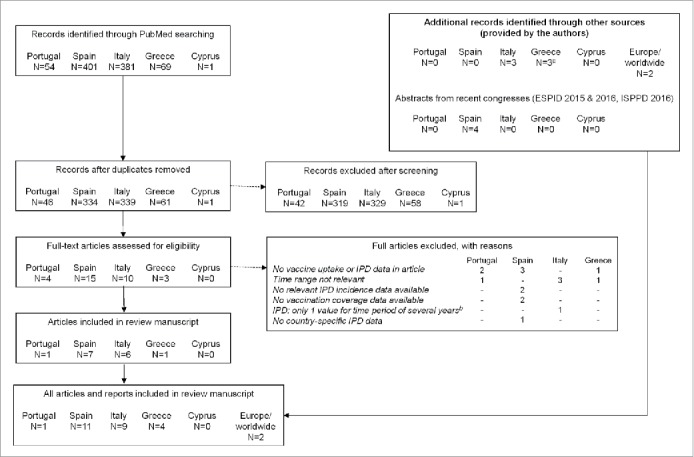
Literature search results. N, number of articles; IPD, invasive pneumococcal disease. The initial screening was done based on the abstracts of the articles. Our literature search used broad keywords and thus returned a fair amount of records; most of these records were excluded because of their lack of relevance to the present review (i.e., no PCV coverage data or IPD incidence within the assessed timeframe). ^a^Includes a peer-reviewed publication published after August 2016, which was proposed during discussion of this review manuscript. ^b^Because we wanted to assess the evolution of IPD incidence over time, manuscripts that provided only 1 incidence estimate covering several years were excluded.

An overview of the literature search is provided in Figure 1; no data were obtained for Cyprus. Table 1 summarizes information about the PCV vaccination programmes and IPD surveillance systems in the Southern European countries. Table 2 presents the PCV vaccination coverage data retrieved. Note that the number of received doses was often not specified, but one can assume that the coverage rate reflects the full schedule if the assessment is being done in 2-year-olds. Nevertheless, one should keep in mind that children may have been partially vaccinated and thus, that not all 2-year-olds will have received the full schedule.

**Table 1.** Pneumococcal conjugate vaccination on national level and IPD surveillance in Southern European countries.

Southern European countries	Introduction of or switch to Extended-valency PCV in NIP	Type of IPD surveillance according to ECDC criteria^50^	IPD surveillance performed by^12^	Immunization schedule
Portugal	July 2015	Not Available	Portuguese PD study group^a^	2, 4 + 12 mo (no catch-up)
Spain	January 2015 (implementation in all regions planned for 2016)	Voluntary, passive	National Centre for Microbiology of Instituto del Salud Carlos III	2, 4 + 12 mo^b^ (no catch-up)
Italy	NIP: 2012 (regional: as of 2010)	Compulsory, comprehensive, passive	Instituto Superiore di Sanità (ISS)	3, 5 + 11–13 mo (no catch-up)
Greece	As of 2010	Voluntary,^c^ comprehensive, passive	National Meningitis Reference Laboratory	2, 4, 6 + 12–15 mo (catch-up for children 18 mo – 5 y)
Cyprus	As of 2010	Voluntary, sentinel, active	Ministry of health	2, 4, 6 + 12 mo

Footnote: mo, months (age); PD, pneumococcal disease; PCV, pneumococcal conjugate vaccine; NIP, national immunisation programme; ECDC, European Centre for Disease Prevention and Control; y, year.

a Portuguese Group for the Study of Streptococcal Infections and the Portuguese Study Group of Invasive Pneumococcal Disease of the Paediatric Infectious Disease Society;

b schedule as foreseen in NIP;

c based on ref. 37 and input from the local author. Of note, Greece and Cyprus earlier had PCV7 introduced in their NIP (in Italy, PCV7 was not in the NIP but was included in several regional vaccination programmes).

**Table 2.** PCV vaccination in Southern European countries.

	Source	Vaccination coverage	Age at assessment	Nr of doses	Source/method of data collection
Portugal	WHO^34^	2015: 80%	NA	1 dose	Official country reported coverage estimates
	VENICE II^12^	2009: 52%	24 months	NS; probably complete schedule^*^	Immunization registry
	Aguiar, 2014^10^	2008: 75%	Up to 2 years	NS; probably complete schedule^*^	NS
		2012: 62%			
Spain^a^	WHO^34^	NA			
	VENICE II^12^	NA			
	Fenoll, 2016^18^	Before 2015: ∼40%	<2 years	NS; probably complete schedule^*^	Data from Intercontinental Marketing Services
*Madrid*	HERACLES^15^^,^^19^	2007-2012: 95%	0–24 months	NS; probably complete schedule^*^	Data from Intercontinental Marketing Services
		2012/13: 82%			
		2013/14: 67%			
		2014/15: 73%			
*Navarre*	Guevara, 2014^22^	2003: 25%	<2 years	Any dose	Regional vaccination register
		2009: 61%			
		2013: 78%			
*Majorca*	Picazo, 2013^24^	2008: 28%	<2 years	Complete schedule	Based on number of vaccine doses sold
		2009: 26%			
		2010: 67%			
Italy	WHO^34^	2007: 55%	12–23 months	3 doses	2007: survey results, 2013: reported data, 2014: coverage reported by national government, 2015: reported administrative data (provisional)
		2013: 87%			
		2014: 87%			
		2015: 89%			
	VENICE II^12^	2008: 55%	12-23 months	NS; probably complete schedule^*^	Cluster-sampling survey
	D'Ancona 2015^25^	2009: 70%	24 months	Complete schedule	Up to 2011 (2005-2009 birth cohorts): survey conducted in 2013 2012 and 2013: Ministry of Health
		2010: 75%			
		2011: 82%			
		2012: 88%			
		2013: 87%			
	Bonanni 2015^5^	2014: 87%	24 months	Complete schedule	Ministry of Health – preliminary data
*Apulia*	D'Ancona 2015^25^	2009: 76%	24 months	Complete schedule	Up to 2011 (2005-2009 birth cohorts): survey conducted in 2013: 2012 and 2013: Ministry of Health
		2010: 81%			
		2011: 93%			
		2012: 95%			
		2013: 93%			
	Fortunato, 2015^30^	2012: 95%	<2 years	Complete schedule	Regional computerised Immunisation Registry
	Martinelli 2014^31^	2013: 93%			
*Sicily*	D'Ancona 2015^25^	2009: 91%	24 months	Complete schedule	Up to 2011 (2005-2009 birth cohorts): survey conducted in 2013: 2012 and 2013: Ministry of Health
		2010: 94%			
		2011: 95%			
		2012: 95%			
		2013: 93%			
	Amodio, 2013^32^	2009: 87%	NS	NS	NS
		2011: 92%			
Greece	WHO^34^	2006-2013: 32%	2006-2011: NS	3 doses	2006-2011: extrapolation
		2014: 96%	2012: 6 years		2012: national government (survey in children 6 years of age)
			2013: NS		2013: national government
			2014: 2–3 years		2014: national government (survey in children 2–3 years of age)
	VENICE II^12^	NA			
	Georgakopoulou 2016^35^	2013: 82%	12 months	3 doses	Cross-sectional nationwide vaccination coverage study

Footnote: NA, not available; NS, not specified; WHO, world health organisation; VENICE, Vaccine European New Integrated Collaboration Effort.

* deduced from the age at which coverage was assessed.

a All Spanish regions are presented in Figure 3.

### Portugal

In Portugal, the decision whether to include a vaccine in the NIP is made nationally by the Ministry of Health, based on the opinion provided by Direção Geral de Saúde and Comissão Técnica de Vacinas. Before July 2015, PCVs were not included in the Portuguese NIP, but were recommended for children and adolescents at risk.^8^ For children who were not at risk, PCVs were not provided by the government but were available on the private market. As of May 2015, it was officially confirmed that a higher-valent PCV (i.e., PCV13) would be part of the NIP for all children born in 2015; it would also be recommended and provided for free to risk groups (children and adults) and would be partially reimbursed for the rest of the population if they have a medical prescription.^9^

**Figure 2.**
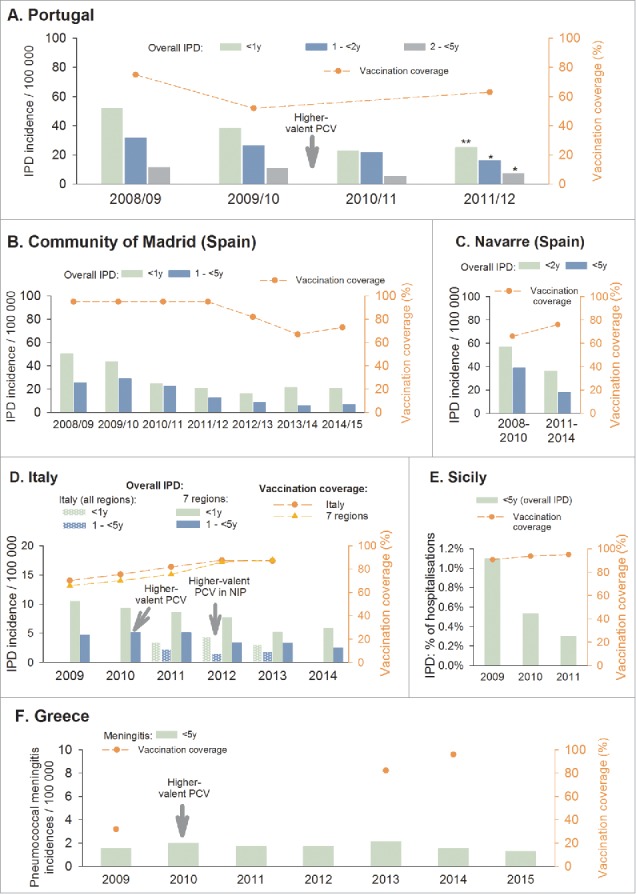
IPD incidence in children less than 5 y old and PCV vaccination coverage. *Footnote: IPD, invasive pneumococcal disease; y, years. The dotted line represents vaccination coverage estimates at the indicated time points. A. Vaccination coverage in children below 2 y of age: 2008 and 2012 estimates were reported by Aguiar et al. 2014,**^10^* *and the 2009 estimate by VENICE (number of doses not specified).**^12^* Statistically significant difference in IPD incidence between 2008/09 and 2011/12: *p = 0.002, **p<0.001. *^10^* *At all time points, PCVs were available only on the private market (not funded by the government). B. IPD and vaccination coverage adapted from Ruiz-Contreras et al, ESPID 2016^15^ and Ruiz-Contreras et al, ISPPD 2016*^19^ *(coverage: children <2 years, number of doses not specified). C. IPD and vaccination coverage (≥ 1 dose) adapted from Guevara et al, Euro Surveillance*.*^23^* *D. National vaccination coverage rates: children 2 y of age, complete schedule (D'Ancona et al, Epidemiol Prev 2015).**^25^* *IPD data from Istituto Superiore di Sanità, 2015*^29^*and D'Ancona et al, Epidemiol Prev 2015.*^25^ *E. IPD data adapted from Amodio et al, Ig Sanita Pubbl 2013*^32^ *and D'Ancona et al, Epidemiol Prev 2015,*^25^ *vaccination coverage (children 2 y of age, complete schedule) from D'Ancona et al, Epidemiol Prev 2015*.^25^ *F. National vaccination coverage for 3 doses: the 2009 coverage estimate is based on extrapolation from data reported by the national government and the 2014 estimate on a survey in children 2-3 y of age (WHO)**^34^**; the 2013 estimate (3 doses) is from a cross-sectional nationwide study*.^35^ *Meningitis data adapted from the National Meningitis Reference Laboratory Data 1993-2015.*^38^

Portugal initially saw a steady increase in uptake of PCV7 in the private market (without reimbursement), reaching 75% of children up to 2 y of age in 2008.^10^ PHiD-CV became available mid-2009 and PCV13 in early 2010; vaccination coverage declined to 63% in children below 2 y of age in 2012.^10^ Another study reported a vaccination coverage of 52% in children 2 y of age in 2009,^11^ based on the data provided in the VENICE II report.^12^ Despite the decline in vaccination coverage, a significant decrease in overall IPD incidence was observed when comparing 2008/09 with 2011/12, in children younger than 5 y (Fig. 2A).^10^

### Spain

In Spain, the decision whether or not to include a vaccine in the NIP is made nationally by the Ministerio de Sanidad, Servicios Sociales e Igualdad. However, the decision of which vaccine will be locally implemented is made by each one of the 19 Autonomous Regions (Comunidades Autonomas - CCAA), explaining why each immunisation program is called a Regional Immunisation Programme. Before January 2015, PCV was not included in the Spanish national immunisation program, except for immunocompromised or high-risk children. However, PCV vaccination was temporarily implemented in 2 autonomous Spanish regions (Galicia and Madrid),^13^ with high coverage rates in those regions (∼100% in Galicia,^14^ and 95% in the Madrid region^15^). In January 2015, the introduction of PCV in the NIP was announced; all 19 Autonomous Regions had until the end of 2016 to implement PCV vaccination.^16^ In Valencia, overall coverage by private funding was 70%; after inclusion of PCV13 in the Valencian Region vaccination program in 2015, vaccination coverage was 94% for the first dose, 89% for the second dose, and 20.4% for a third dose (of note, official recommendation is 2 primary doses plus booster, not a 3+1 schedule).^17^

**Figure 3.**
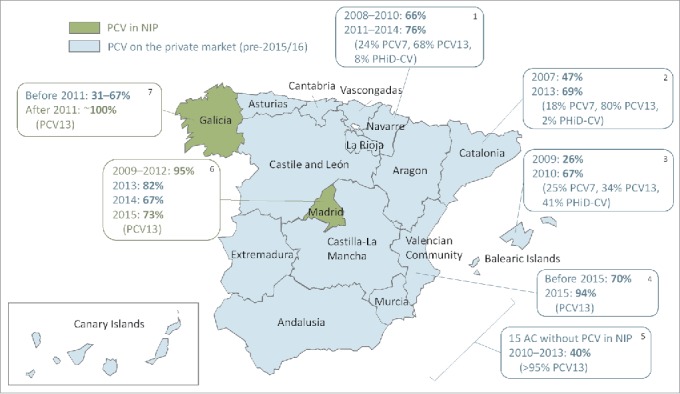
PCV coverage in the different Spanish regions. PCV, pneumococcal conjugate vaccine; NIP, national immunization program; AC, autonomous communities. Data adapted from: 1. Navarra: Guevara et al. Euro Surveill 2016 ^23^; 2. Cataluña: del Amo et al. PLoS ONE 2016 ^63^; 3. Mallorca: Picazo et al. BMC Infect Dis 2013 ^24^; 4. Valencia: Boone et al. ESPID 2016 ^17^; 5. 15 regions: Fenoll et al. Vaccine 2015 ^18^; 6. Madrid: Ruiz-Contreras ISPPD 2016 ^19^; 7. Galicia: Rivero-Calle ISPPD 2016.^14^

In the regions that had not included PCVs in their vaccination calendar before 2015, the estimated coverage (based on a 3-dose schedule) was around 40% in 2010–2013. These estimates were based on data from Intercontinental Marketing Services; vaccination coverage was calculated by dividing the total number of doses by the number of children under 2 y of age.^18^ Coverage rates varied across the different regions (Fig. 3).

In the Madrid region (6 million inhabitants), PCV13 was introduced from May 2010 to May 2012 into the Madrid Regional Immunisation Programme for children below 2 y of age, replacing PCV7, which had been included since 2006. Vaccination coverage in children up to 2 y of age, based on data from Intercontinental Marketing Services, was 95% in 2007–2012, but after funding was ceased in May 2012, vaccination rates dropped to 82% in 2012/13 and 67% in 2013/14.^15^^,^^19^ After re-introduction of PCV13 in March 2015, uptake was 73%.^19^ A considerable decrease in overall IPD incidence was observed after the introduction of PCV13. However, after vaccination coverage dropped to 67%, an increase in overall IPD incidence was observed in children below 2 y of age, from 16.2 per 100,000 in 2012/13 to 21.6 in 2013/14, which slightly improved again to 20.7 in 2014/15. Children between 2 and 5 y of age maintained the decrease in overall IPD incidence rate as this group was still well vaccinated (Fig. 2B).^15^^,^^19^ Note that the IPD incidence rates from the most recent years are preliminary data as presented at the ESPID 2015 and ISPPD 2016 conferences; incidence rates up to 2012 have already been published.^20^^,^^21^

In the Navarre region (640 000 inhabitants), PCV7 became available on the private market in June 2001, PHiD-CV was introduced in November 2009, and PCV13 replaced PCV7 in June 2010. The percentage of children younger than 2 y who had received at least 1 PCV dose was 25% at the end of 2003, increased to 61% at the end of 2009, and to 78% at the end of 2013.^22^ When assessing several years pooled together, the percentage of children who had received at least 1 PCV dose was 66% in 2008–2010, and 76% in 2011–2014.^23^ The IPD incidence in children younger than 2 y and in children younger than 5 y decreased over time (Fig. 2C).^23^ Guevara et al. had previously reported a decrease of 69% in IPD incidence in children younger than 5, from 60.7 cases per 100 000 in 2004–2009 to 18.7 in 2010–2013.^22^

In the Island of Majorca (900,000 inhabitants), PCVs were not included in the childhood immunisation program until 2015. Coverage of the full vaccination schedule (3+1 doses) in children below 2 y of age, based on the number of PCV doses sold, was low in 2008 (28%) and 2009 (26%) but increased to 67% in 2010 after the launch of higher-valent PCVs. In children below 1 y of age, IPD incidence rates per 100 000 were 11.65 (1 case) in 2008, 10.54 (1 case) in 2009, and 47.34 (4 cases) in 2010. In children between 1 and 2 y of age, IPD incidence rates were 82.71 (8 cases) in 2008, 94.18 (9 cases) in 2009, and 19.31 (2 cases) in 2010.^24^ The low numbers of reported cases in these age groups make it difficult to discern trends in IPD incidence over time; the apparent increase in IPD in children below 1 y of age was not statistically significant, while the decrease in the 1–2 y age group was significant (p<0.05 between 2010 and 2009).^24^

### Italy

In Italy, PCV was included in the NIP in 2012, but several regions had already implemented PCVs in regional vaccination programmes, starting from 2005. The average Italian vaccination coverage in terms of children that received a complete PCV schedule by 2 y of age, progressively increased from 46% in 2007 to 88% in 2012.^25^ In 2012−2014, overall PCV coverage in Italy decreased by 0.4%.^5^

As of 2013 (birth cohorts 2011 and 2012), vaccination coverage is routinely being collected by the Ministry of Health; estimates for earlier birth cohorts were collected through a dedicated web survey. Vaccination coverage varies considerably between different regions, with estimates for 2013 ranging from 45% in Calabria to 99% in Basilicata.^25^ For 2014, estimates ranged from 77% for Campania to 99% for Basilicata (the only region to meet the 95% coverage rate target).^5^ A complete overview of the coverage estimates in the various regions for 2015 is available on the website of the Istituto Superiore di Sanità.^26^

The number of IPD cases in Italy tended to decrease between 2009 and 2013, but stabilised in 2012–2013 in children between 1 and 4 y of age.^27^^,^^28^ IPD incidence rates in Italy for 2011 until 2013 are shown in Fig. 2D.^29^ While the ‘malattie batteriche invasive’ (MIB) surveillance system collects notifications from all 21 Italian regions, many of them report a very low number of cases and under-reporting is suspected for some areas. Therefore, the IPD incidence was also assessed separately for the 7 Italian regions with highest aptitude for reporting (Piemonte, PA Trento, PA Bolzano, Lombardia, Veneto, Friuli-Venezia Giulia, and Emilia-Romagna).^25^ Similar trends were observed as for the whole of Italy, but with higher incidence rates (Fig. 2D).^25^

In the Apulia region (4 million inhabitants), PCV7 was introduced in the regional immunisation schedule in January 2006 and replaced by PCV13 in May 2010. Coverage for full vaccination schedule in children below 2 y of age was estimated to be 95% in 2012 (2010 birth cohort; PCV7/PCV13) and 93% in 2013 (2011 birth cohort).^25^^,^^30^ In total, between May 2010 and January 2013, 4 laboratory-confirmed IPD cases were identified in children younger than 5 y (1 case in 2010, 2 cases in 2011, and 1 case 2012).^31^ An assessment of hospital records extracted from the regional discharge registry revealed 6 IPD cases in the same period (1 case in 2010, 2 in 2011, and 3 in 2012).^30^

In Sicily, infants are routinely vaccinated with PCV since 2004, with vaccination coverage increasing from 87% in 2009 to 92% in 2011 (age and number of doses not specified).^32^ D'Ancona et al. reported vaccination coverage of 91% in 2009, 95% in 2011 and 2012, and 93% in 2013 (complete schedule by 2 y of age).^25^ Active surveillance of IPD (PneumoNet) was performed in Sicily from 2009 to 2011. Based on hospital discharge data of the 9 regional participating hospitals, the proportion of hospitalizations for IPD decreased in children younger than 5 y (Fig. 2E).^32^

In the Veneto region, vaccination coverage increased from 83.7% in 2007 to 90.3% in 2011, and decreased thereafter to 87.9% in 2012 and 88.4% in 2013.^25^ An active surveillance of invasive bacterial diseases has been implemented in this region since 2007. IPD notification rates in children younger than 5 y decreased from 6.2 per 100 000 in 2007–2010 to 2.9 per 100,000 in 2011–2014.^33^

### Greece

Coverage rates in Greece for 3 PCV doses were estimated at 32% in 2009 (based on extrapolation from data reported by the national government) and 96% in 2014 (survey in children 2–3 y of age).^34^ In a cross-sectional vaccination coverage study in 2013 assessing preschool children attending nurseries-kindergartens, 82.3% of the studied population had received 3 PCV doses by 12 months, while 62.3% received the 4^th^ dose by 24 months and 76.2% by 30 months, indicating a delay for the booster dose.^35^ In a case-control study, for a control group of 42 children aged 24–59 months and born after higher-valent PCV implementation, 85.7% were found to have received the complete 3+1 immunization series.^36^

In Greece, meningitis surveillance is performed by the National Meningitis Reference Laboratory; IPD surveillance is not actively performed. The Laboratory collaborates with all National Hospitals, who voluntarily send their samples for typing and recording. In 2014, the Reference Laboratory reported to receive samples for typing corresponding to approximately 80–90% of all cases of meningitis or septicaemia.^37^ For typing and recording of meningitis cases, the Laboratory asks the hospitals to send cerebrospinal fluid samples. The annual incidence of pneumococcal meningitis in Greek children below 5 y of age seemed to be generally stable between 2009 and 2013, while there was a trend toward decreasing incidences in the most recent years (Fig. 2F).^38^

Pneumococcal community-acquired pneumonia (CAP) with empyema also seemed to remain stable between 2008 and 2016,^36^ although this is the experience of a single tertiary care center. A total of 30 CAP cases with empyema of any etiology occurred: 10 in 2008/09, 4 in 2010/11, 7 in 2012/12 and 9 in 2014/Jan 2016. Of the 27 cases in which the pathogen was identified, 19 were *S. pneumoniae* (4 in 2008/09, 4 in 2010/11, 6 in 2012/13, and 5 in 2014/Jan 2016). Between 2008 and 2011, among the 13 children who were aged 2 months or older, 9 (69.2%) were fully vaccinated with PCV7 and 2 (15.4%) partially; none of these 11 children had received any dose of higher-valent PCV. Between January 2012 and January 2016, 13 (81.2%) of the 16 children with empyema were fully vaccinated with a PCV.^36^

### Discussion

This review alerts about potential dangers of a suboptimal vaccination coverage that seems to be present in some regions of the Southern European countries (Portugal, Spain, Italy, and Greece) within the studied period. The World Health Organization (WHO) currently recommends administration of 3 PCV doses, in a schedule of either 3 primary doses or 2 primary doses plus a booster.^39^ The Global Vaccine Action Plan (GVAP WHO 2011–2020 framework approved by the World Health Assembly in May 2012 to achieve the Decade of Vaccines vision by delivering universal access to immunization) aims to reach vaccination coverage of at least 90% nationally and at least 80% in every district by 2020 for vaccines in national programmes, including PCV.^40^^,^^41^

We would like to re-emphasize the need for better vaccination coverage and IPD incidence data; the variable and fragmented nature of the data we found makes it difficult to conclude on trends in vaccination coverage as well as their impact on overall IPD incidence. Moreover, for Cyprus, no relevant data were found within the search period of this study.

### Limitations regarding vaccination coverage data

Only partial vaccination coverage data are publicly available in Southern Europe at both national and regional levels, with a lack of annual data (Table 2). Moreover, estimation ranges often vary considerably between different sources. There are several reasons for the fragmented nature and variability of the data, including differences in timing of the reports, and the way vaccination coverage rates are collected and calculated. Data collected via administrative methods or surveys may have potential inaccuracies in their numerator or denominator; rates will also vary when obtained via calculating the number of doses that are distributed, versus assessing how many doses were actually administered. Another important shortcoming is the fact that many reports and publications do not specify if the reported vaccination coverage rates represent the administration of at least 1 dose, 2 doses, or the complete schedule (Table 2). As incomplete vaccination may occur relatively often, the reported or projected vaccination coverage rate may be questionable if a full vaccination schedule is assumed.

The latest available VENICE II report from 2012^12^ mentions that 2 of the 5 Southern European countries (Portugal and Italy) have adopted a nationwide immunisation registry. Before implementation of PCVs in the Portuguese NIP, pneumococcal vaccination coverage was not assessed regularly.^12^ In Italy, pneumococcal vaccination coverage is assessed yearly via regional-level computerised systems (15/21 regions are fully computerised, 5 regions are partially computerised and 1 does not use a computerised register).^42^^,^^43^ Since 2013, regional data have been collected by the Ministry of Health and made publically available.^27^^,^^28^ A computerised vaccinations register is available in the Spanish regions of Murcia and Navarre (2 out of 19 regions).^43^ In Greece, vaccination coverage data are collected approximately every 5 years, using a survey-based methodology.^44^^,^^45^ In the WHO database,^34^ vaccination coverage data are unavailable for certain countries (e.g. Spain).

Thus, difficulties in obtaining accurate vaccination coverage estimations in Southern Europe remain a concern, despite the knowledge that assessment of vaccination coverage is an essential component of vaccine program monitoring and evaluation. Accurate assessment of vaccination coverage is of particular importance when vaccination is expected to provide herd protection against vaccine types in other age groups not eligible for vaccination, even though protection against overall IPD (net benefit) is inconsistent and varies among countries in persons above the age of 65 y.^46^

### Limitations regarding IPD data

The type of IPD surveillance varies between the different Southern European countries, with voluntary vs. compulsory surveillance and active vs. passive surveillance. Definitions of IPD may also vary, and for Greece, only pneumococcal meningitis rates were available while IPD incidences were not reported. In 2010, Hanquet and colleagues proposed to initially focus on the collection and analysis of meningitis data from all countries as a minimum common data set, as one of the considerations for establishing a uniform European IPD surveillance system.^47^ In some countries however, PCV use had variable influence on the rate of pneumococcal meningitis; while a decrease in pneumococcal meningitis morbidity and mortality rates was reported in Brazil after introduction of PHiD-CV,^48^ another study reported large confidence intervals,^49^ and in several countries, only a slight decrease in pneumococcal meningitis cases after PCV13 implementation was observed.^50^ This variability could increase the difficulty to estimate the potential impact of vaccination on pneumococcal disease, when based solely on meningitis data.

Because variation in inter-country IPD incidence may be related to differences in national surveillance systems and diagnostic practices (especially regarding laboratory confirmation), no comparison between countries could be made in this study.

While most reports and manuscripts present annual data, this is not always the case (e.g., Navarre),^22^ and some reports present only the number of IPD cases without calculating the IPD incidence (e.g., Italy before 2011).^27^

Of note, some publications and reports also provide details on VT and NVT IPD, and we could have focused on these serotypes in terms of interpretation of potential trends of incidences. However, as quoted by Hausdorff et al.^1^ ‘the overall impact of a given PCV formulation on IPD cannot be predicted solely on the basis of its serotype content, as there can be vaccine-specific differences in serotype efficacy, in cross-protection and perhaps even herd protection and serotype replacement, not to mention other pathogens.’ Therefore, focusing on trends of IPD caused by VT and NVT in relation to vaccination coverage would not reflect the full benefit of PCVs seen a potential bigger influence of vaccination coverage in VT and VRT and their cross-protection as well as the complex dynamics of VT, VRTs and NVT (replacement phenomena). For this reason, and considering that overall IPD (i.e., the net benefit) is of high interest with regard to public health and would ultimately better describe the impact of PCVs, we have decided to evaluate overall IPD incidences.

For completeness however, we also assessed IPD caused by VT with respective available vaccination coverage data, as shown in Supplementary Fig. 1. Trends for VT IPD were similar to those for overall IPD, with decreasing incidences when coverage improved, and a trend toward increased VT IPD incidence when a decrease in coverage occurred (e.g., 2012/13 vs 2013/14 in children younger than 1 y of age in Madrid^15^) (Supplementary Fig. 1). Trends are less clear with regards to NVT IPD and vaccination coverage. As observed in many countries, NVT IPD tended to increase over time in Italy^10^ and Navarre (Spain)^23^ (Supplementary Fig. 2). In Portugal, a similar trend toward increased NVT IPD was observed in the group aged 2 up to 5 years, whereas NVT IPD incidences remained stable in the younger age groups^10^ (Supplementary Fig. 2).

### Inability to make a link between vaccination coverage and IPD data sets

Few reports assess both vaccination coverage rates and IPD incidence, and the above-mentioned limitations make it difficult to link vaccination coverage and IPD data sets from different studies. Thus, the impact of potentially decreasing vaccination coverage was difficult to assess in most regions. However, in the Madrid region, both vaccination coverage and IPD incidence rates were collected and the drop in vaccination coverage after funding was ceased was shown to correlate with an increased IPD incidence in children younger than 2 years,^15^ illustrating that a correlation can be made when sufficiently accurate data are available. The incidence of bacteraemic community-acquired pneumonia in children up to 14 y of age was also shown to increase in the 2 y after PCV was withdrawn from the Madrid vaccination program, from 2.1/100,000 children in 2012 to 5.4/100,000 children in 2014.^51^ There was also an increase in complications of pneumococcal bacteraemia in the same age group after 2012, although no increase in complications due to PCV13 serotypes was observed.^52^

### Data for the most recent years are not yet available

For many of the regions, data were only available until 2014, which is relatively close to the start of the EU financial crisis (as of end of 2009) to see a potential impact on vaccination coverage or IPD incidence. Moreover, PCVs have been adopted in the NIP of Portugal and Spain from 2015 onwards. Higher coverage rates may be expected in the future, similarly to other NIP vaccines in these countries. Therefore, a close follow-up of the overall IPD incidence and vaccination coverage in these 5 Southern European countries should be maintained and improved in order to evaluate the efficiency of higher-valent PCV vaccination programmes and to follow up a potential impact of vaccination coverage on unvaccinated populations.

### European-wide efforts toward vaccination coverage assessment

European-wide vaccination coverage data collection is performed by WHO/UNICEF via the Joint Reporting Form on Immunisation. At the moment, WHO/Europe's centralised information system for infectious diseases (CISID) remains the main source for assessing vaccination coverage in the EU, but data on PCV vaccination is at the moment not available in the CISID database. Vaccination coverage assessment in Europe and potential issues were previously described.^53^

As discussed earlier, the surveillance systems for invasive bacterial disease currently remain very diverse across Europe, which complicates the comparison of data between countries. IPD surveillance could be improved by using standard methods and by providing laboratory confirmation of reported IPD cases. Alternatively, clinically suspected IPD cases (i.e., not culture-confirmed) could be collected through related ICD-10 codes from national infectious registers. ECDC is making considerable efforts to collect data from all EU countries. These data are published in an annual epidemiological report and in a dedicated report, but there is a delay in availability of the yearly data. Since August 2012, ECDC has been funding SpID-net (Streptococcus pneumoniae Invasive Disease network), a pilot project aiming to set up active surveillance of IPD in Europe to monitor the impact of PCV vaccination programmes; this project should overtake the limitation of the various national surveillance systems.^54^ The latest data of SpID-net-2, collected at 13 surveillance sites in Europe (including 3 sites in Spain), were presented at the ISPPD-10 Congress in June 2016. After 5 y of PCV10(PHiD-CV)/PCV13 vaccination, IPD incidence (all serotypes) in children younger than 5 y decreased by 50%, when annual IPD incidence after PCV10/13 introduction (2010–2014) by age group and serotype category was compared with the average IPD incidence during the PCV7 period.^55^

### Vaccination coverage in Southern Europe

Despite the gaps in the available data, it can be concluded that vaccination coverage in many Southern European regions needs to be improved in order to meet the goals set by GVAP/WHO (2011–2020). In Italy, there is a very large variability in vaccination coverage between the different regions with some regions obtaining sufficiently high vaccination rates while others are clearly lacking.^5^^,^^25^

While many European countries currently recommend a 2+1 vaccination schedule, a 3+1 schedule is still recommended by the government in Greece. However, the recommended 3 primary doses are often not reached. In a prospective survey conducted in 2009–2011 in 1667 children aged 6 months in Athens, 77% of the children had received ≥ 1 dose of PCV, and 79% had received ≥ 2 doses at 12 months, but only 43% of 12-month old children had received 3 PCV doses.^30^ A prospective longitudinal study conducted in 2010-2011 among toddlers (2–5 y of age) attending day care centers in Greece (Athens and Viotia) also indicated that incomplete vaccination was relatively frequent: while 97% of the children had been vaccinated with ≥ 1 dose of PCV7, 76% had received ≥ 3 doses of PCV7.^56^ Moreover, only 143 of the 225 vaccinated toddlers (64%) had initiated PCV immunisation at the age of 2-6 months, suggesting that also a delay of vaccination may be an issue.^56^ A cross-sectional study from 2013 showed that 82% of the studied children had received 3 doses by the age of 12 months, but also observed a reduced coverage with the 4^th^ dose and a delayed vaccination completion.^35^

Important initiatives have occurred as Portugal has introduced PCV13 in its NIP in 2015 and Spain will in 2016 also have PCVs implemented in all autonomous regions. Consequently, coverage uptake is expected to reach high levels in both countries.

### Potential for increased impact on IPD with higher levels of vaccination coverage

After implementation of higher-valent PCVs and despite suboptimal vaccination coverage, IPD incidence tended to decrease in the Southern European countries; the direct and indirect (VTs) impact could likely be even higher with a better vaccination coverage. Even though there are no published national data from PCV effectiveness or impact studies in these Southern European countries, overall IPD impact of both higher-valent PCVs was observed in different populations as discussed below. With a sufficiently high level of vaccination coverage and an IPD epidemiology close to the serogroups contained in the higher-valent PCVs, a similar range of impact on IPD should be expected in Southern Europe.

There are no double-blind randomized controlled trials (DBRCTs) with PCV13, but 2 DBRCTs, in Finland (FinIP) and in Argentina, Colombia and Panama (COMPAS), reported vaccine effectiveness/efficacy (VE) of PHiD-CV against culture-confirmed overall IPD. VE of 93% (95% confidence interval [CI]: 75-99) was observed in FinIP for the 3+1 and 2+1 schedules combined^57^ while VE of 67% (95% CI: 22–86) was observed in COMPAS.^58^ Effectiveness against IPD was also shown in post-marketing studies for both PHiD-CV and PVC13. In a matched case-control study in Brazil, the adjusted effectiveness of an age-appropriate PHiD-CV schedule was 84% (95% CI: 66-92) against vaccine-serotype IPD.^59^ In a population-based study in Finland, the overall IPD rate among vaccine-eligible children was reduced by 80% (95% CI: 72-85), while the reduction in vaccine-type IPD was 92% (95% CI: 86-95).^60^ For PCV13, vaccine effectiveness against all-cause IPD was 60.2% (95% CI: 46.8-70.3) in a matched case-control study in the USA,^49^ while in England and Wales, incidences of overall IPD in children younger than 2 y in 2013/14 decreased by 56% (15.63 vs 6.85 per 100 000; IRR 0.44, 95% CI: 0.43-0.47) when compared with the pre-PCV7 baseline.^61^ In Canada (Quebec), VE against vaccine-type IPD in children having received at least 1 dose was 97% (95% CI: 84-99) for PHiD-CV and 86% (95% CI 62-95) for PCV13, while VE against overall IPD was 72% (95% CI: 46-85) for PHiD-CV and 66% (95% CI: 29-83) for PCV13. No substantial difference was observed between PHiD-CV, PCV13 or a mixed schedule with both vaccines.^2^

Palmu et al.^62^ have proposed to assess clinically suspected IPD on the basis of International Classification of Diseases, 10th Revision, diagnoses compatible with IPD (A40.3/ B95.3/G00.1/M00.1) and unspecified sepsis (A40.9/A41.9/A49.9/G00/G00.9/I30.1/M00/M00.9/B95.5), by evaluating hospitals’ inpatient and outpatient discharge notifications of non-laboratory-confirmed (i.e., clinically suspected) IPD or unspecified sepsis collected from the national Care Register. This provides an interesting opportunity for surveillance, when keeping in mind the potential difficulties linked to surveillance and sensitivity of culture-confirmed IPD. Their vaccine-probe analysis was the first report showing the effect of PCVs on clinically suspected IPD. The absolute rate reduction was markedly higher compared with laboratory-confirmed IPD, which implies low sensitivity of the laboratory-based case definitions and subsequently higher public health effect of PCVs against IPD than previously estimated.^62^ From a public health standpoint, this vaccine-probe analysis could be considered when pondering on IPD and PCV benefits in children in Southern Europe.

So far, no trends of increasing overall IPD incidence have yet been observed even with suboptimal vaccination coverage. However, a higher impact of vaccination could be expected with higher vaccination coverage, and the level of vaccination coverage – much more than the choice of which vaccine to use – will drive impact on pneumococcal disease. To improve vaccination coverage, different strategies could be implemented at various levels (parents, physicians, government).

### Study limitations

There is a lack of published articles in peer-reviewed journals to confirm the data from gray literature (e.g., non-peer-reviewed reports), which could potentially compromise the validity of the reported findings. Moreover, the reports were not designed to assemble data as we have done in the current manuscript. Finally, more information on vaccination coverage or IPD rates might be available at the national level, but without being publicly or at least timely available.

### Conclusion

As efforts are already deployed at the European and national level, the aim should be to focus on a continued IPD surveillance with timely publicly available reports and adequate national vaccination registers to follow up trends in vaccination coverage, allowing the evaluation of PCV implementation in the NIP. In addition to culture-confirmed IPD surveillance, assessing national registers for clinically suspected IPD as a manner to quantify impact on overall IPD would allow to show higher public health effect of pneumococcal conjugate vaccines. In order to improve vaccination coverage data, vaccination documentation should be collected in a complete manner with age of the assessed group and the indication if the rates refer to 1st dose, >1 dose, 2 doses or full schedule (3 or 4 doses) and indicating whenever possible the vaccination timing to detect potential delays.

### Methodology

We searched PubMed for manuscripts published between 1 January 2009 and 4 August 2016. The following keywords were used: “pneumococcal conjugate vaccine coverage," “vaccination coverage," and “invasive pneumococcal disease;" each of the search terms were combined with each selected/targeted country (Portugal, Spain, Italy, Greece, and Cyprus). Manuscripts in English as well as in the local languages were included. Relevant manuscripts had to contain data about IPD incidence, or about vaccination coverage with higher-valent PCVs. Only regions with data available on both IPD incidence and vaccination coverage (even if from different sources) were included in the manuscript. The target population was children younger than 5 y of age. Studies containing only data for the year 2009 and earlier were excluded, as the higher-valent PCVs were only introduced as of 2009. Of note, vaccination coverage data for the years 2009 and 2010 might contain data for PCV7 or for a mix of PCV7 and PHiD-CV/PCV13.

In addition to the PubMed search, authors from each country provided additional non-peer-reviewed reports and conference abstracts or other gray literature (outside of commercial or academic publishing channels) from national or regional public health institutions involved in IPD surveillance.

### Trademark statement

Prevenar/Prevnar and Prevenar 13/Prevnar 13 are trademarks of Wyeth Pharmaceuticals Inc., a subsidiary of Pfizer Inc. Synflorix is a trademark of the GSK group of companies.

## Supplementary Material

Supplemental_material.zip
