# Supplementary material for: A reflection on invasive pneumococcal disease and pneumococcal conjugate vaccination coverage in children in Southern Europe (2009–2016)
Source: Hum Vaccin Immunother. 2016 Dec 20;13(6):1242–53. doi: 10.1080/21645515.2016.1263409 (PMC5489303; doi:10.1080/21645515.2016.1263409)
Supplement: Supplemental_material.zip [file khvi-13-06-1263409-s001.zip › Figure S2.docx]

**Supplementary figure 2. Overall and non-vaccine-type IPD incidence in children less than 5 years old and PCV vaccination coverage**

*Footnote: IPD, invasive pneumococcal disease; y, years. The dotted line represents vaccination coverage estimates at the indicated time points.*

*A. Vaccination overage in children below 2 years of age: 2008 and 2012 estimates were reported by Aguiar et al. 2014,*[*^1^*](#_ENREF_1) *and the 2009 estimate by VENICE (number of doses not specified).*[*^2^*](#_ENREF_2) *Statistically significant difference in IPD incidence between 2008/09 and 2011/12: *p=0.002, **p<0.001.* [*^1^*](#_ENREF_1) *At all time points, PCVs were available only on the private market (not funded by the government).*

*B. National vaccination coverage rates: children 2 years of age, complete schedule (D’Ancona et al, Epidemiol Prev 2015).*[*^3^*](#_ENREF_3) *IPD data from Istituto Superiore di Sanità, 2015*[*^4^*](#_ENREF_4)*and D'Ancona et al, Epidemiol Prev 2015*[*^3^*](#_ENREF_3)*.*

*C. IPD and vaccination coverage (≥1 dose) adapted from Guevara et al, Euro Surveillance*[*^5^*](#_ENREF_5)*.*

**References**

1. Aguiar SI, Brito MJ, Horacio AN, Lopes JP, Ramirez M, Melo-Cristino J, Portuguese Group for the Study of Streptococcal I, Portuguese Study Group of Invasive Pneumococcal Disease of the Paediatric Infectious Disease S. Decreasing incidence and changes in serotype distribution of invasive pneumococcal disease in persons aged under 18 years since introduction of 10-valent and 13-valent conjugate vaccines in Portugal, July 2008 to June 2012. Euro Surveill 2014; 19: 20750.

2. VENICE II. Impact of childhood pneumococcal vaccination programmes and activities for pneumococcal vaccines in the EU and EEA \EFTA countries. 2012. Available from: <http://venice.cineca.org/VENICE_Survey_PNC_1_2012-02-24.pdf>

3. D'Ancona F, Caporali MG, Del Manso M, Giambi C, Camilli R, D'Ambrosio F, Del Grosso M, Iannazzo S, Rizzuto E, Pantosti A. Invasive pneumococcal disease in children and adults in seven Italian regions after the introduction of the conjugate vaccine, 2008-2014. Epidemiol Prev 2015; 39: 134-8.

4. Istituto Superiore di Sanità. Dati di sorveglianza delle malattie batteriche invasive aggiornati al 23 marzo 2015. 2015. Available from: <http://www.iss.it/binary/mabi/cont/Report_MBI_20150323_V8.pdf>

5. Guevara M, Barricarte A, Torroba L, Herranz M, Gil-Setas A, Gil F, Bernaola E, Ezpeleta C, Castilla J, Working Group for Surveillance of the Pneumococcal Invasive Disease in N. Direct, indirect and total effects of 13-valent pneumococcal conjugate vaccination on invasive pneumococcal disease in children in Navarra, Spain, 2001 to 2014: cohort and case-control study. Euro Surveill 2016; 21.
