# Supplementary material for: A reflection on invasive pneumococcal disease and pneumococcal conjugate vaccination coverage in children in Southern Europe (2009–2016)
Source: Hum Vaccin Immunother. 2016 Dec 20;13(6):1242–53. doi: 10.1080/21645515.2016.1263409 (PMC5489303; doi:10.1080/21645515.2016.1263409)
Supplement: Supplemental_material.zip [file khvi-13-06-1263409-s001.zip › Figure S1.docx]

**Supplementary figure 1. Overall and vaccine-type IPD incidence in children less than 5 years old and PCV vaccination coverage**

*Footnote: IPD, invasive pneumococcal disease; y, years. The dotted line represents vaccination coverage estimates at the indicated time points.*

*A. Vaccination overage in children below 2 years of age: 2008 and 2012 estimates were reported by Aguiar et al. 2014,*[*^1^*](#_ENREF_1) *and the 2009 estimate by VENICE (number of doses not specified).*[*^2^*](#_ENREF_2) *Statistically significant difference in IPD incidence between 2008/09 and 2011/12: *p=0.002, **p<0.001.*[*^1^*](#_ENREF_1) *At all time points, PCVs were available only on the private market (not funded by the government).*

*B. IPD and vaccination coverage adapted from Ruiz-Contreras et al, ESPID 2016*[*^3^*](#_ENREF_3) *and Ruiz-Contreras et al, ISPPD 2016*[*^4^*](#_ENREF_4) *(coverage: children <2 years, number of doses not specified).*

*C. IPD and vaccination coverage (≥1 dose) adapted from Guevara et al, Euro Surveillance*[*^5^*](#_ENREF_5)*.*

*D. National vaccination coverage rates: children 2 years of age, complete schedule (D’Ancona et al, Epidemiol Prev 2015).*[*^6^*](#_ENREF_6) *IPD data from Istituto Superiore di Sanità, 2015*[*^7^*](#_ENREF_7) *and D'Ancona et al, Epidemiol Prev 2015*[*^6^*](#_ENREF_6)*.*

**References**

1. Aguiar SI, Brito MJ, Horacio AN, Lopes JP, Ramirez M, Melo-Cristino J, Portuguese Group for the Study of Streptococcal I, Portuguese Study Group of Invasive Pneumococcal Disease of the Paediatric Infectious Disease S. Decreasing incidence and changes in serotype distribution of invasive pneumococcal disease in persons aged under 18 years since introduction of 10-valent and 13-valent conjugate vaccines in Portugal, July 2008 to June 2012. Euro Surveill 2014; 19: 20750.

2. VENICE II. Impact of childhood pneumococcal vaccination programmes and activities for pneumococcal vaccines in the EU and EEA \EFTA countries. 2012. Available from: <http://venice.cineca.org/VENICE_Survey_PNC_1_2012-02-24.pdf>

3. Ruiz-Contreras J, Picazo J, Casado-Flores J, Negreira S, Baquero F, Hernández-Sampelayo T, Otheo E, Del Amo M. IPD evolution in Madrid children by age group before and after the drop of PCV13 vaccination rates: Heracles study (2007-14). ESPID, 2015.

4. Ruiz-Contreras J, Picazo J, Casado-Flores J, Negreira S, Baquero F, Hernández-Sampelayo T, Otheo E, Del Amo M. IPD incidence rates and serotype evolution following reduction of PCV13 vaccination uptake after switching from universal to private funding in Madrid, Spain: HERACLES study (2007-15). 10th International Symposium on Pneumococci and Pneumococcal Diseases (ISPPD10) Glasgow, Scotland, 2016.

5. Guevara M, Barricarte A, Torroba L, Herranz M, Gil-Setas A, Gil F, Bernaola E, Ezpeleta C, Castilla J, Working Group for Surveillance of the Pneumococcal Invasive Disease in N. Direct, indirect and total effects of 13-valent pneumococcal conjugate vaccination on invasive pneumococcal disease in children in Navarra, Spain, 2001 to 2014: cohort and case-control study. Euro Surveill 2016; 21.

6. D'Ancona F, Caporali MG, Del Manso M, Giambi C, Camilli R, D'Ambrosio F, Del Grosso M, Iannazzo S, Rizzuto E, Pantosti A. Invasive pneumococcal disease in children and adults in seven Italian regions after the introduction of the conjugate vaccine, 2008-2014. Epidemiol Prev 2015; 39: 134-8.

7. Istituto Superiore di Sanità. Dati di sorveglianza delle malattie batteriche invasive aggiornati al 23 marzo 2015. 2015. Available from: <http://www.iss.it/binary/mabi/cont/Report_MBI_20150323_V8.pdf>
